# The Development of a Scoring System for Acute Variceal Bleeding Risk in Cirrhotic Patients in Emergency Departments

**DOI:** 10.3390/life16040665

**Published:** 2026-04-14

**Authors:** Wei-Yu Lin, Chia-Lung Kao, Yi-Jia Liu, Peng-Peng Chang, Ming-Yuan Hong

**Affiliations:** 1 Department of Emergency Medicine, National Cheng Kung University Hospital, College of Medicine, National Cheng Kung University, Tainan 704, Taiwan; 2 Emergency Department, Tainan Hospital, Ministry of Health and Welfare, Tainan 70043, Taiwan; 3 The Center for Quantitative Sciences, Clinical Medicine Research Center, National Cheng Kung University Hospital, Tainan 701, Taiwan

**Keywords:** liver cirrhosis, gastroesophageal variceal bleeding, gastrointestinal hemorrhage, prothrombin time international normalized ratio, end-stage renal disease

## Abstract

Objective: To identify independent clinical predictors of acute variceal bleeding (AVB) in cirrhotic patients and to develop a rapid, non-invasive scoring system to facilitate objective risk stratification and resource prioritization in emergency departments (EDs). Methods: This retrospective study focused on the development of a scoring system based on the international normalized ratio of prothrombin time (PT INR) and end-stage renal disease (ESRD) hemodialysis (HD) status to aid in predicting acute variceal bleeding. Results: In our study involving 319 patients, we report an association between a prolonged PT INR (OR 1.73, 95% CI 1.03–2.91; *p* = 0.038) and the absence of ESRD (*p* < 0.001) and an increased risk of variceal bleeding. The resulting risk-scoring system, while preliminary, ranges from 2 to 14 points and shows promise, with an AUC of 0.89 suggesting its utility in emergency departments. Conclusions: This scoring system, although in its early stages, may be a beneficial tool in emergency care for patients with cirrhosis. Its practicality and potential efficiency could aid in better patient management. However, broader validation in diverse clinical settings is essential to confirm its applicability and effectiveness.

## 1. Introduction

Gastroesophageal variceal bleeding is an important medical emergency that can lead to significant morbidity and mortality [1]. Globally, liver cirrhosis represents a major health burden, accounting for approximately 2.4% of annual deaths, and serves as the common pathway for various chronic liver diseases. The progression to portal hypertension often leads to severe complications, with variceal hemorrhage being the most lethal.

However, distinguishing variceal hemorrhage from other sources of gastrointestinal bleeding solely on the basis of early clinical presentation remains a significant challenge. In emergency departments, early detection of acute bleeding is a priority to enable physicians to identify urgent bleeding patients, shorten the time to clinical intervention, and reduce morbidity and associated costs.

Portal hypertension leads to gastroesophageal variceal bleeding, which is a gastrointestinal emergency and one of the major causes of death in patients with liver cirrhosis [2]. The clinical treatment goals of variceal bleeding include achieving hemostasis and avoiding complications related to bleeding [2,3]. Endoscopic therapy, including band ligation and sclerotherapy, is the treatment option for controlling gastroesophageal variceal bleeding. Recent research has further refined these techniques; for example, randomized controlled trials have compared the efficacy of combined scleroligation versus band ligation alone, highlighting the continuous evolution of endoscopic strategies to improve patient outcomes [4].

The Baveno VII Consensus Workshop suggests that patients with features of cirrhosis and upper gastrointestinal bleeding should be treated with early endoscopy within 12 h of presentation [5]. Acute variceal bleeding is associated with mortality in cirrhotic patients, and early recognition that enables prompt treatment is of clinical importance [3]. Despite advances in care, the prognosis remains guarded, with AVB having a high in-hospital mortality rate (ranging from 7% to 15% in the literature and 7.9% in our study). This underscores the necessity for tools that facilitate the Baveno VII recommendation of early endoscopy within 12 h of presentation.

A prospective multicenter study indicated that the probability of bleeding from esophageal varices is related to the Child–Pugh score, the size of the varices, and the degree of red wale markings [6]. Several studies have indicated that the location, size, and appearance of the varices and the variceal pressure might be potential risk factors for variceal hemorrhage [7,8,9,10]. However, most of these factors should be confirmed with endoscopy [11]. Previous studies have investigated potential risk factors and analyzed them with traditional regression methods. However, these studies might have overlooked associated risk factors that were not considered in the regression model [12,13,14].

Current methodologies, such as endoscopic procedures and complex scoring systems such as the Child-Pugh and MELD scores, primarily assess the prognosis of liver cirrhosis rather than the immediate risk of acute variceal bleeding. This highlights the following critical knowledge gap: while existing models predict patients who have cirrhosis or who might die within six months, there is a lack of practical, bedside tools to predict patients who are bleeding before an endoscopy can be performed. Most traditional risk factors—such as variceal size or red wale markings—cannot be assessed until the patient is already on the procedure table, leaving a diagnostic void during the initial hours of emergency triage.

While these methods are valuable in their context, they may not be ideal in urgent care settings such as emergency departments, where rapid, specific risk evaluation for variceal bleeding is crucial. Our previous study examined the role of ABO blood types in mortality and rebleeding risk in patients with acute variceal hemorrhage [15]. We found no significant differences in outcomes between the blood type O and non-O groups (*p* = 0.532), indicating that fixed genetic traits are inadequate for immediate risk assessment. This underscores the need to identify dynamic clinical and laboratory parameters that can provide actionable information during the crucial initial hours of emergency triage.

This study aimed to develop a simplified, noninvasive scoring system to quickly identify and triage cirrhotic patients at high risk for acute variceal bleeding in the emergency department, improving resource allocation for early endoscopy.

## 2. Materials and Methods

### 2.1. Study Population and Data Collection

This retrospective study was conducted at National Cheng Kung University Hospital, from January 2014 to August 2019. We initially screened 828 ED visits of cirrhotic patients who presented with nontraumatic gastrointestinal bleeding, of which 649 visits involved patients who underwent endoscopy to confirm the bleeding source. Following the exclusion of visits for patients under 18 years of age, those with noncirrhotic diagnoses, or those with incomplete medical records, the final analysis included 600 ED visits from 319 unique patients.

Because each presentation to the emergency department requires an independent clinical triage decision, the unit of analysis was the individual ED visit. To account for the statistical dependency arising from repeated observations within the same individual, we utilized a generalized linear mixed-effects model (GLMER), designating the individual patient as a random effect.

Acute variceal bleeding was defined as symptoms such as hematemesis and melena, which were confirmed by endoscopy. Factors assessed included vital signs, laboratory data, hepatoma, hepatitis B virus (HBV) infection, hepatitis C virus (HCV) infection and ESRD, defined as patients with kidney failure requiring a regular course of long-term hemodialysis), hypertension (HTN), and diabetes mellitus (DM) status. The Child–Pugh score was used to gauge liver cirrhosis severity. Univariate and multivariate analyses revealed significant risk factors.

Two trained senior nurses extracted the demographic data, medication history, laboratory data, and endoscopy findings from medical records, with accuracy verified by senior emergency physicians. AVB was defined as symptoms of hematemesis or melena and was confirmed via endoscopy. The key factors assessed included vital signs, hepatitis status, and ESRD, the latter of which was defined as kidney failure requiring a regular course of long-term hemodialysis.

### 2.2. Statistical Analysis and Study Design

The study followed a systematic two-stage approach to model development and evaluation. To address the statistical dependency arising from repeated observations within the same individual (multiple visits per patient), we utilized a generalized linear mixed-effects model (GLMER), designating the individual patient as a random effect.

Stage 1: Variable identification and scoring system development. We performed univariate and multivariate analyses using a generalized linear mixed-effects model (GLMER). The GLMER model was implemented in the R environment (version 4.1.0; R Foundation for Statistical Computing, Vienna, Austria), utilizing the glmer function within the lme4 package. By treating the individual patient as a random effect, we accounted for the statistical dependence among 600 visits from 319 unique patients. On the basis of the coefficients derived from the multivariate model, we identified independent predictors (PT INR and ESRD status) and assigned weighted points to establish a 2 to 14 points scoring system (range 2–14) and a corresponding clinical nomogram for bedside application.

Stage 2: Performance evaluation and clinical utility. Predictive performance was primarily evaluated by discrimination, assessed using the area under the receiver operating characteristic (ROC) curve (AUC). In addition, the calculated scores were descriptively linked to the observed probabilities of acute variceal bleeding (AVB) within the study population to illustrate risk stratification across score levels. Clinical utility was considered based on the tool’s potential to facilitate rapid triage and prioritize high-risk patients for early endoscopy. This two-stage approach allowed us to transition from raw clinical data to a validated, practical tool for emergency triage.

To account for the statistical dependency arising from repeated observations within the same individual (600 visits from 319 patients), we utilized a generalized linear mixed-effects model (GLMER). In this model, we specified a random intercept for the individual patient to account for inherent baseline differences in bleeding risk across the sample. We focused on the fixed effects of PT INR and ESRD status to identify stable predictors for our bedside scoring system. The model employed an independent covariance structure, ensuring that the estimated standard errors and *p*-values were appropriately adjusted for the nested data structure without over-parameterizing the model.

To ensure the reliability and stability of this model, we followed the events per variable (EPV) criterion. This rule ensures that a model has sufficient clinical events to justify the “predictors” used, thereby preventing overfitting. A standard requirement in medical modeling is to have at least 10 events per predictor variable. With 279 confirmed AVB events and two primary predictors in our final model (PT INR and ESRD status), the EPV ratio was 139.5, which substantially exceeds the commonly recommended minimum threshold. This suggests that the model was developed under a favorable sample size condition and may reduce the risk of overfitting.

The final risk model and predictive accuracy were validated through ROC curves, with the AUC and accuracy calculated for the developed scoring system.

Ethical approval for the study was obtained from the Ethics Review Board of National Cheng Kung University Hospital, with patient consent waived because of its retrospective nature.

## 3. Results

### 3.1. Demographic Data

**Figure 1:**
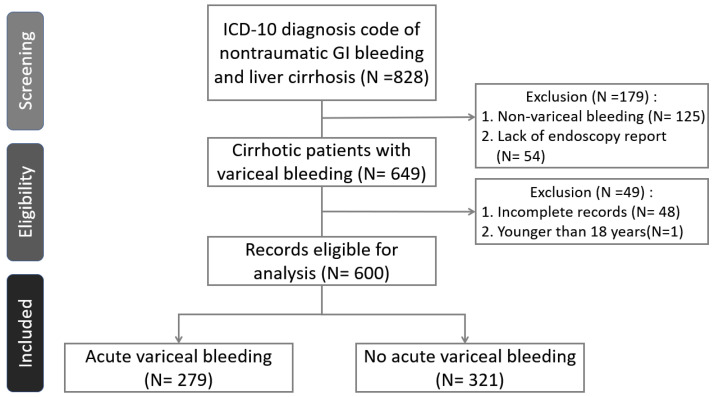
Flowchart of participant selection.

A total of 828 ED visits were made by patients with an ICD-10 diagnosis code of liver cirrhosis and nontraumatic GI bleeding underwent screening, of which patients in 649 ED visits received endoscopy (Figure 1). Overall, 49 patient visits were excluded because of incomplete data or an age younger than 18 years, and 319 eligible patients and 600 visits were included in the analysis. All patients received a proton pump inhibitor (PPI) and a vasoactive drug (terlipressin acetate) on admission to the ED and prior to endoscopy. The baseline demographic and clinical characteristics and endoscopic findings are presented in Table 1. Among the 600 visits of patients with liver cirrhosis who visited the ED because of acute GI bleeding, 279 were confirmed to have acute variceal bleeding, and 321 had no acute variceal bleeding while they underwent endoscopy. The prevalence rate of ESRD in patients without acute variceal bleeding was 8%, which was higher than the prevalence rate (2%) in patients with acute variceal bleeding. The prevalence rate of HBV/HCV in the acute variceal bleeding group was 68%, which was higher than the prevalence rate (58%) in the no acute variceal bleeding group. With respect to the initial data at admission to the ED, the eGFR of patients in the acute variceal bleeding group was 71.23 ± 25.18 mL/min/1.73 m^2^, which was higher than that of patients without acute variceal bleeding (64.27 ± 28.32). Compared with those in patients without acute variceal bleeding, the prothrombin time and INR in patients with acute variceal bleeding were greater. With respect to endoscopic treatment, more patients received ligation, histoacryl or cyanoacrylate injection, and S-B tubes in the acute variceal bleeding group than in the group without acute variceal bleeding.

### 3.2. Model Establishment

There were four factors with significant differences according to the 95% confidence interval (CI) during univariate analysis, namely, ESRD, estimated glomerular filtration rate (eGFR), prothrombin time (PT), and the international normalized ratio (INR). Kendall’s concordance coefficient plot was subsequently used to evaluate the correlation of these significant variables. The PT and PT INR were strongly correlated (0.924). The PT INR was adopted because it is a normalized and corrected index that is a more objective measure of coagulation. ESRD and the eGFR were moderately correlated (0.320), and we fit each variable in the individual model because the clinical characteristics of ESRD patients who received hemodialysis may differ from those of patients who did not receive hemodialysis. We selected the two most significant factors, PT INR and ESRD, as predicting factors to fit the first model through the GLMER [16,17,18,19]. Both factors had a significant influence, while each effect was fixed; the odds ratio of the PT INR was 1.73 (95% confidence interval (CI), 1.03–2.91), and the odds ratio of ESRD was 0.33 (95% CI, 0.12–0.92). The area under the curve (AUC) of the receiver operating characteristic (ROC) curve was 0.887, and the accuracy was 81.9% (sensitivity 73.5%, specificity 89.1%).

### 3.3. Establishment of the Scoring System

**Figure 2:**
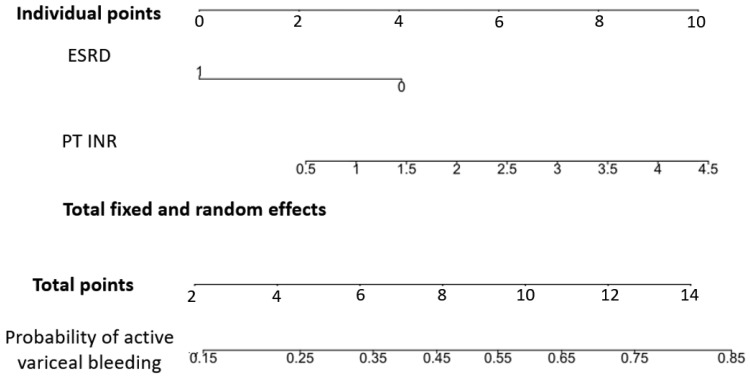
Nomogram and prediction score for the probability of acute variceal bleeding. PT: prothrombin time; INR: international normalized ratio; ESRD: end-stage renal disease.

After adjusting for fixed and random effects, we used a nomogram of the odds ratios of the factors in the established models to calculate the corresponding points of the prediction scores (Figure 2). The number of effect points ranged from 0 to 10, which corresponded to the odds ratio of each factor. In the model, the PT INR (Table 2) ranged from 0.5 to 4.5, which corresponded to 2 points to 10 points; thus, when the PT INR increased by 0.5 units, the number of points increased by 1. A patient with ESRD with hemodialysis corresponds to 0 points, and a patient without ESRD corresponds to 4 points. The final scoring system provides a total score ranging from 2 to 14, representing 13 distinct risk levels. These scores correspond to a calculated probability of acute variceal bleeding, varying from 14% to 83% (Table 3). This range allows for granular risk stratification in the emergency department setting.

## 4. Discussion

This retrospective study involved 319 eligible patients with 600 visits who presented with acute gastroesophageal variceal bleeding that was confirmed and treated by endoscopy. Patients with a prolonged PT INR had a greater risk of acute variceal bleeding (OR 1.73, 95% CI, 1.03–2.91). On the basis of the results, we developed a 2 to 14 points risk-scoring system to predict acute variceal bleeding, with a higher number of points corresponding to a higher risk of acute variceal bleeding. The AUC of this scoring system was 0.887, and the accuracy was 81.9%. Our study supports the notion that a prolonged PT INR is associated with a higher risk of acute variceal bleeding. This study proposes a scoring system that may be useful for evaluating acute variceal bleeding. The score provides a simple bedside framework for early risk stratification. By incorporating two clinical variables, it may help emergency physicians rapidly identify patients at higher probability of active variceal bleeding and facilitate earlier preparation for urgent endoscopic evaluation.

The results of this study suggest that the PT INR might serve as a potential predictor of acute variceal bleeding in cirrhotic patients. The PT INR, which reflects liver function and the severity of decompensation, is a predictor of acute variceal bleeding. While some studies affirm its significance in predicting rebleeding risk, others report no significant prolongation of the PT INR among cirrhotic patients with bleeding varices, underscoring the need for further investigation [20,21,22,22]. According to the results of our study, cirrhotic patients who presented to the ED with a clinical presentation of upper GI bleeding had a greater incidence of acute variceal bleeding if the PT INR increased. The degree of prolonged PT INR was associated with the probability of acute variceal bleeding in our study. The increase in the PT INR correlated with the likelihood of acute variceal bleeding in a dose-dependent manner. This finding implies that elevated PT INR values may indicate a more significant degree of liver dysfunction, consequently heightening the risk of acute variceal bleeding.

This study indicates that considering ESRD hemodialysis status alongside the PT INR might be relevant for assessing the risk of acute variceal bleeding, although further research is needed. We observed that hemodialysis patients with ESRD are less likely to present with acute variceal bleeding, potentially because of the effect of hemodialysis on reducing portal pressure and, consequently, bleeding risk. This findings indicate that ESRD patients on regular hemodialysis might have a lower likelihood of acute variceal bleeding, although the relationship is complex and may require further study for a comprehensive understanding. Theoretically, the bleeding alterations of end-stage renal disease make patients prone to bleeding complications. The pathogenesis of hemorrhagic diatheses in ESRD patients is multifactorial and includes impaired platelet adhesiveness, abnormal platelet endothelial interactions, and decreased coagulation factors [23,24]. A previous study revealed that patients who received hemodialysis had a higher risk of developing nonvariceal or lower gastrointestinal bleeding [25,26]. In terms of variceal bleeding, it is essential to note that varices are portosystemic collaterals formed when preexisting vascular channels are dilated because of portal hypertension. Portal hypertension is evaluated by measuring the hepatic venous pressure gradient. Among liver fibrosis patients on dialysis, the hepatic venous pressure gradient was lower than that in patients who did not receive dialysis [27]. Hemodialysis may reduce portal pressure in cirrhotic patients; consequently, a reduction in portal pressure may decrease the risk of acute variceal bleeding [28]. Such a discrepancy between studies has raised questions regarding whether patients on hemodialysis for ESRD are prone to variceal bleeding. Despite the importance of bleeding complications, studies on the impact of hemodialysis on cirrhosis patients with gastroesophageal variceal bleeding are still lacking. Our results revealed that patients who regularly received hemodialysis had a lower risk than patients who did not. However, more clinical studies are needed for confirmation.

Unlike these traditional models, which assess overall liver disease severity, our system is specifically calibrated for the acute bleeding phenotype by focusing on objective variables: PT INR and ESRD status. This approach fills a critical diagnostic void in the emergency department, where identifying high-risk patients early is vital. Given that delayed endoscopy is a recognized risk factor for in-hospital mortality in cirrhotic patients [29], our scale facilitates the ‘priority thinking’ necessary to meet the 12 h endoscopy window recommended by Baveno VII. By simplifying risk prediction, this system shows promise for broader application in clinical settings to improve resource allocation and patient outcomes.

In the context of risk assessment, our 2 to 14-point scoring system prioritizes objectivity and speed, offering a more nuanced and pragmatic alternative to traditional prognostic tools and complex diagnostic models. While established systems like the Child-Pugh and MELD scores are informative regarding liver disease severity, they often include parameters such as ascites and hepatic encephalopathy, which are subject to significant inter-observer variability and can be confounded by hypotension or hemorrhagic shock in acute emergency settings. By focusing exclusively on PT INR—a standardized laboratory parameter—and ESRD status—a binary clinical characteristic—our model eliminates these subjective ‘gray areas’ and the need for time-consuming bedside ultrasonography as required by other assessment methods [30]. The scoring system proposed in this study was designed to support rapid bedside risk stratification in the emergency department. Although the variables included in the scoring system overlap with those used in existing scoring systems, they are integrated in a simplified and context-specific manner to facilitate more rapid identification of patients at higher probability of active variceal bleeding and to support earlier preparation for urgent endoscopic evaluation. Furthermore, unlike complex ‘black box’ artificial neural networks (ANNs) that may achieve high AUCs but lack transparency, our scale is readily calculable at the bedside in seconds. This approach facilitates a rapid, reproducible triage tool that aligns with the exigent workflow of busy emergency departments, ensuring that resource prioritization is based on transparent, data-driven parameters.

As research on noninvasive predictors of variceal complications expands, various methodologies have emerged, from scoring systems utilizing biochemical and ultrasound parameters in critical care [31] to complex artificial neural networks (ANNs) that integrate over a dozen risk factors to achieve high predictive accuracy (e.g., AUC 0.959) [32]. However, our approach differs significantly by focusing specifically on the immediate risk of active bleeding during the critical window of emergency department triage. While high-parameter models or those requiring specialized imaging may offer impressive statistical performance, they are often impractical in time-sensitive, high-pressure settings. By providing a streamlined, 2-variable method that does not rely on specialized equipment or ‘black box’ algorithms, our scoring system offers the bedside practicality essential for rapid clinical decision-making in urgent emergency care.

The value of this scoring system lies in its ability to facilitate rapid resource prioritization. While all gastrointestinal (GI) bleeding patients require urgent care, AVB carries a distinct in-hospital mortality rate of 7.9% in our cohort. Because delayed endoscopy is a recognized risk factor for death in these specific patients, clinicians must possess the “priority thinking” to distinguish a variceal phenotype from more stable sources before the procedure begins.

## 5. Strengths and Limitations

The primary strength of this study lies in its rigorous methodology. By employing a generalized linear mixed-effects model (GLMER), we effectively addressed the correlation among 600 visits from 319 unique patients, ensuring statistical independence. Additionally, the EPV ratio was 139.5, based on 279 bleeding events and 2 predictors, which supports the adequacy of the sample size for model development and may have reduced the risk of overfitting, although it does not replace formal internal validation.

Our study has several limitations, emphasizing the need for caution in interpreting the results. As a single-center retrospective analysis, it may introduce inherent biases, and our prediction model awaits external validation to confirm its broader applicability and generalizability. The modest sample size could also predispose our findings to statistical errors. Selection bias, imprecise data on nonendoscopic treatments, and potential socioeconomic disparities further highlight the caution required in generalizing our results. The study lacked a validation cohort to further assess model performance. Although the EPV ratio was high (139.5; 279 bleeding events for 2 predictors), a high EPV is not a substitute for formal internal validation. Therefore, the absence of bootstrapping or cross-validation should be considered a limitation. While the model demonstrated good discriminatory ability, its calibration and real-world clinical utility remain to be established in future studies. Further research with external validation cohorts and more comprehensive model evaluation is warranted to confirm the robustness and generalizability of these findings.

## 6. Conclusions

The primary objective of this study was to develop a simplified, objective scoring system to predict the immediate risk of AVB among cirrhotic patients in emergency departments.

To address the urgent need for efficient patient triage in overcrowded emergency departments, our study introduces a modest scoring system that leverages the PT INR and ESRD hemodialysis status for the rapid risk assessment of acute variceal bleeding in cirrhotic patients. This approach aims to enhance resource allocations and patient outcomes by prioritizing patients who need immediate endoscopic therapy. By acknowledging the pressing challenges of limited resources, this system represents a step toward improving emergency care efficiency.

This study has two key implications. First, it shifts the focus from long-term prognosis, typically measured by MELD or Child-Pugh scores, to immediate preendoscopic risk assessment in emergency situations. Second, it suggests that regular hemodialysis may protect against AVB, likely by reducing portal pressure, warranting further investigation. While this system improves the efficiency of emergency care and resource allocation for early endoscopy, its effectiveness needs to be validated across diverse clinical settings.

## Figures and Tables

**Table 1:** Demographics of the cirrhotic patients with gastroesophageal varices.

	Acute Variceal Bleeding (N = 279)	No Acute Variceal Bleeding (N = 321)	Mean/Proportion Difference (95% CI)
Characteristics			
Age (years)	61.93 ± 12.07	63.95 ± 14.35	0.095 (−0.030, 0.002) ^†^
Male sex—no. (%)	197 (71.0)	223 (69.0)	0.894 (−0.423, 0.485) ^‡^
Coexisting diseases—no. (%)			
Hypertension	69 (25)	74 (23)	0.473 (−0.307, 0.662) ^‡^
Diabetes mellitus	108 (39)	103 (31)	0.178 (−0.134, 0.722) ^‡^
End-stage renal disease	6 (2)	24 (8)	0.007 (−2.502, −0.389) ^‡^
Hepatoma	110 (39)	108 (34)	0.275 (−0.196, 0.657) ^‡^
HBV	97 (35)	99 (31)	0.563 (−0.314, 0.577) ^‡^
HCV	104 (37)	100 (31)	0.056 (−0.012, 0.896) ^‡^
HBV/HCV	191 (68)	187 (58)	0.102 (0.022, 0.182) ^‡^
Alcoholism	103 (37)	106 (33)	0.328 (−0.223, 0.669) ^‡^
Initial data on ED admission			
Systolic blood pressure (mmHg)	123.24 ± 26.07	126.10 ± 24.82	0.271 (−0.012, 0.003) ^†^
Systolic blood pressure < 90 mmHg—no. (%)	49 (18)	43 (14)	0.315 (−0.229, 0.813
Mean blood pressure (mmHg)	89.00 ± 19.13	90.67 ± 17.96	0.400 (−0.015, 0.006) ^†^
Mean blood pressure < 65 mmHg—no. (%)	24 (9)	16 (5)	0.200 (−0.260, 1.243) ^‡^
Heart rate (beats/min)	100.17 ± 20.17	97.07 ± 20.75	0.078 (−0.001, 0.018) ^†^
Heart rate > 100 beats/min—no. (%)	141 (51)	147 (46)	0.367 (−0.209, 0.565) ^‡^
Shock index > 1—no. (%)	50 (18)	56 (17)	0.727 (−0.598, 0.417) ^‡^
Hemoglobin level (13.3–17.2 g/dL)	8.63 ± 2.21	9.06 ± 2.45	0.067 (−0.162, 0.006) ^†^
Platelet count (143–149 10^3^/µL)	116.28 ± 66.44	122.36 ± 87.02	0.354 (−0.004, 0.001) ^†^
Creatine (0.70–1.20 mg/dL)	1.38 ± 3.35	1.94 ± 6.58	0.303 (−0.086, 0.027) ^†^
Alanine aminotransferase (≤50 U/L)	45.37 ± 51.92	42.50 ± 47.55	0.540 (−0.003, 0.005) ^†^
eGFR (≥60 mL/min/1.73 m^2^)	71.23 ± 25.18	64.27 ± 28.32	0.010 (0.002, 0.017) ^†^
Prothrombin time (9.4–12.5 s, INR)	16.65 ± 5.41 (1.50 ± 0.42)	15.62 ± 4.14 (1.41 ± 0.33)	0.037 (0.003, 0.089) ^†^ (0.008) (0.198, 1.289) ^†^
Activated partial thromboplastin time (29.3–40.1 s)	34.43 ± 8.51	34.92 ± 8.65	0.558 (−0.030, 0.016)
Child–Pugh Score (Class)—no. (%)			
5 to 6 (A)	93(33)	122(38)	ref ^¶^
7 to 9 (B)	132(47)	149(46)	0.389 (−0.246, 0.632) ^¶^
10 to 15 (C)	54(19)	50(16)	0.180 (−0.185, 0.985) ^¶^
Endoscopic treatment			
Door to EGD time (hours)	3.24 ± 4.61	3.35 ± 3.20	0.754 (−0.071, 0.051) ^†^
Number who received ligation (%)	255 (91.4)	47 (14.6)	0.768 (0.713, 0.822) ^‡^
Argon plasma coagulation (%)	11 (3.9)	0 (0)	NA
Histoacryl or cyanoacrylate injection (%)	28 (10)	14 (4.4)	0.057 (0.012, 0.102) ^‡^
S-B tube (%)	18 (6.5)	4 (1.2)	0.052 (0.017, 0.087) ^‡^
Outcomes			
Admission duration (days)	7.84 ± 7.66	7.91 ± 10.97	0.943 (−0.019, 0.021) ^†^
In-hospital mortality (%)	22 (7.9)	22 (6.9)	0.567 (−0.512, 0.936) ^‡^

^†^ Mean difference presented with 95% confidence interval. ^‡^ Differences between two independent proportions are presented with 95% confidence intervals. ^¶^ odds ratio, 5 to 6 (A) was defined as the reference group; HBV: hepatitis B virus; HCV: hepatitis C virus; INR: international normalized ratio; eGFR: estimated glomerular filtration rate.

**Table 2:** PT INR score corresponding to ESRD.

Points	PT INR	ESRD
0		Yes
2	0.5	
3	1.0	
4	1.5	No
5	2.0	
6	2.5	
7	3.0	
8	3.5	
9	4.0	
10	4.5	

PT: prothrombin time; INR: international normalized ratio; ESRD: end-stage renal disease.

**Table 3:** Prediction of acute variceal bleeding versus the calculated score.

Total Points	Probability of Acute Variceal Bleeding (%)
2	14
3	18
4	22
5	27
6	33
7	38
8	47
9	52
10	60
11	67
12	72
13	77
14	83

## Data Availability

The data are unavailable because of privacy or ethical restrictions.

## Abbreviations

ED	emergency department
ESRD	end-stage renal disease
GI	gastrointestinal
CPS	Child–Pugh score
CPC	Child–Pugh class
HTN	hypertension
DM	diabetes mellitus
GLMMs	generalized linear mixed-effects models
GLMs	generalized linear models
LMMs	linear mixed-effects models
PPI	proton pump inhibitor
ALT	alanine aminotransferase
APTT	activated partial thromboplastin time
eGFR	estimated glomerular filtration rate
PT	prothrombin time
INR	international normalized ratio
AUC	area under the curve
ROC	receiver operating characteristic
